# Clinical Use of Diuretics in Heart Failure, Cirrhosis, and Nephrotic Syndrome

**DOI:** 10.1155/2015/975934

**Published:** 2015-07-29

**Authors:** Ahmed Hassaan Qavi, Rida Kamal, Robert W. Schrier

**Affiliations:** ^1^Shifa College of Medicine, Shifa Tameer-e-Millat University, Pitras Bukhari Road, Sector H-8/4, Islamabad 44000, Pakistan; ^2^Division of Renal Diseases and Hypertension, University of Colorado School of Medicine, 12700 East 19th Avenue C281, Research Building 2, Room 7001, Aurora, CO 80045, USA

## Abstract

Diuretics play significant role in pharmacology and treatment options in medicine. This paper aims to review and evaluate the clinical use of diuretics in conditions that lead to fluid overload in the body such as cardiac failure, cirrhosis, and nephrotic syndrome. To know the principles of treatment it is essential to understand the underlying pathophysiological mechanisms that cause the need of diuresis in the human body. Various classes of diuretics exist, each having a unique mode of action. A systemic approach for management is recommended based on the current guidelines, starting from thiazides and proceeding to loop diuretics. The first condition for discussion in the paper is cardiac failure. Treatment of ascites in liver cirrhosis with spironolactone as the primary agent is highlighted with further therapeutic options. Lastly, management choices for nephrotic syndrome are discussed and recommended beginning from basic sodium restriction to combined diuretic therapies. Major side effects are discussed.

## 1. Background

Choosing the suitable use of diuretics in patients with heart failure, nephrotic syndrome and cirrhosis requires an understanding of the pathophysiology of these edematous conditions. These diseases lead to sodium and water retention in patients, causing detrimental effects in their morbidity and mortality. Heart failure decreases cardiac output and cirrhosis causes progressive systemic arterial vasodilation, which eventually leads to ascites [1]. Nephrotic syndrome causes retention through defective glomerular barriers, induction of the distal nephron and altered capillary permeability [2]. It also leads to hypoalbuminemia, which decreases plasma oncotic pressure, thereby indirectly causing edema [3].

The body fluid volume regulation hypothesis suggests a common circulation pathway for the three disorders [4]. According to this, the underfilling due to low cardiac output or peripheral vasodilation leads to activation of sympathetic nervous system and nonosmotic arginine vasopressin release. Consequently, diminished water and sodium delivery at collecting duct sites in addition to renal adrenergic activity induces renin angiotensin aldosterone system, which enhances tubular reabsorption [1, 4–6].

The cortical collecting tubules are the primary site to contribute to the edema formation in nephrotic syndrome. These are primarily made up of principal and intercalated cells which function to reabsorb sodium and water and excrete potassium. Sodium retention is caused primarily by transcriptional induction of Na/K/ATPase pump. This activation is independent of aldosterone and vasopressin [7]. The electrochemical gradient setup by the Na/K/ATPase pump drives sodium through the apical membranes containing epithelial sodium channels (ENaC). Defective glomerular filtration barrier leaks plasma proteases such as plasmin, prostasin, and kallikrein that cause proteolytic activation of ENaC [8, 9]. Enhanced sodium retention through the aforementioned mechanisms along with proteinuria and hypoalbuminemia via impaired glomerular filtration barrier leads to asymmetrical extracellular volume expansion [2, 9].

As described previously, two pathophysiological processes cause edema formation. Firstly, shifts in capillary pressures promote movement of fluid from the vascular compartment into the interstitium. Secondly, the kidneys cause retention of sodium and water [10]. Consequently, there is marked expansion of the total extracellular volume with plasma volume kept close to normal levels. Clinically these events have great significance. Tissue perfusion is returned to normal through appropriate compensation at the price of expanding the degree of edema in most edematous disorders that cause water and sodium retention [10]. Diuretic therapy drains the edema fluid causing recovery from symptoms due to edema but infrequently results in a decrease in tissue perfusion. On the other hand, primary renal dysfunction leads to inappropriate renal fluid retention where both plasma and interstitial volumes are swelled. Hence, diuretic therapy may not cause any significantly adverse effects as superfluous fluid is excreted [10].

The pharmacology of the various classes of diuretics is important to know for clinical application. All classes of diuretics have different mechanisms of action; however various forms of diuretics from one class have similar pharmacological characteristics [11]. For instance, since all loop diuretics operate similarly, addition of another loop diuretic after one with appropriate dosage failing to show response is not warranted. Instead, a combination therapy with administration of different classes of diuretic is recommended [11, 12].

**Figure 1:**
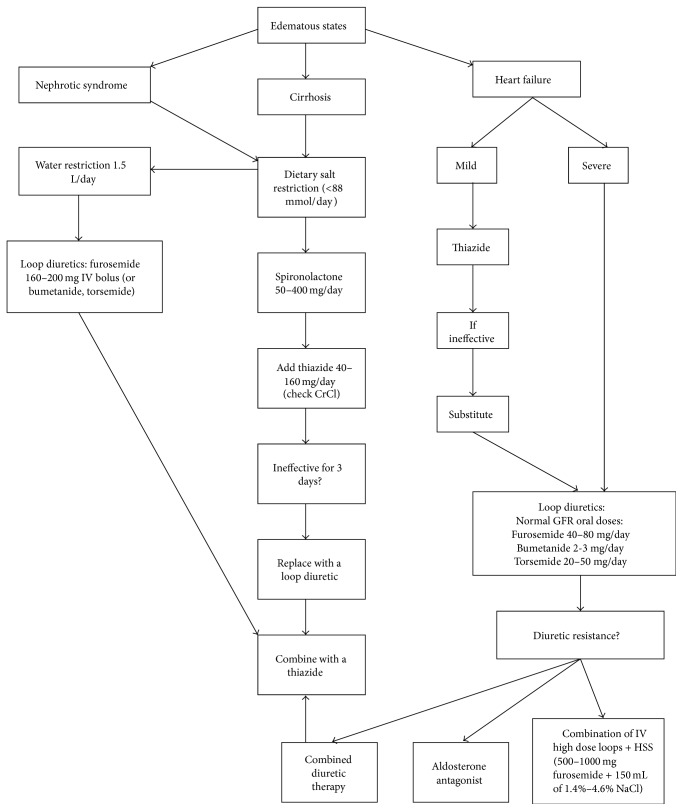
Management of edematous states with diuretics. Abbreviations: HSS: hypertonic saline solution [11, 12, 24, 25, 30–33].

Thiazide diuretics work by blocking the sodium-chloride transporter [12] and loop diuretics act by inhibiting the sodium-potassium-chloride pump in the thick ascending limb of the loop of Henle [13]. Amiloride and triamterene block apical sodium channels in the distal nephron [14, 15]. All diuretics but spironolactone reach these luminal transport sites through the tubular fluid. Spironolactone competitively binds receptors at the aldosterone-dependent sodium-potassium exchange site in the distal convoluted renal tubule. Except osmotic diuretics, all diuretics are actively secreted into the urine by proximal tubule cells. Loops, thiazides, and acetazolamide are secreted through the organic-acid pathway while amiloride and triamterene are secreted through the organic-base pathway [12, 14, 15]. These drugs escape ultrafiltration at the glomerulus due to their high protein binding, more than 95% [11, 12].  Figure 1 outlines the basic management strategies employed in the three main edematous conditions.

## 2. Use of Diuretics in Heart Failure

Heart failure is the foremost cause of morbidity among the elderly Americans. It accounts for more than 1 million hospital admissions annually in the US [16]. After hospitalization, 50% of heart failure patients are readmitted to hospitals within 6 months and 25–30% expire at 1 year [17]. Numerous clinical trials have all failed to deduce a universal drug therapy strategy to treat acute heart failure by decreasing mortality or rehospitalization rates [18]. The Acute Heart Failure Registry (ADHERE), comprising of over 105, 000 hospitalized patients, showed 90% of them being treated with intravenous loop diuretics and 30% showed resistance to diuretic therapy [19]. These patients were suffering from signs and symptoms that included breathlessness (89%), pulmonary rales (67%), and peripheral edema (66%) [19].

Diuretics are well established as the first-line therapy for heart failure patients with congestion [20]. A meta-analysis assessing the benefits of diuretics in chronic heart failure showed a decrease in mortality (3 trials, 202 patients) and worsening heart failure (2 trials, 169 patients) in patients compared to placebo. A few clinical trials (4 trials, 169 patients) also demonstrated that diuretics improved exercise tolerance in patients with chronic heart failure compared to active controls [20]. Diuretics have also established their superiority over device-based strategies. In a randomized controlled trial (RCT) involving 188 hospitalized patients with acute decompensated heart failure, poor renal function, and persistent congestion, treatment with intravenous diuretics was compared to ultrafiltration. Diuretics proved to be more efficacious in the preservation of renal function and had lesser adverse effects than ultrafiltration. The serious adverse effects compared were renal and heart failure, anemia, thrombocytopenia, and gastrointestinal hemorrhage [21].

Mild congestive heart failure is initially managed with a thiazide diuretic [11]. However, loop diuretics (e.g., furosemide, torsemide, or bumetanide) are the principal drugs used in the treatment of heart failure [22]. Severe heart failure causes decrease in the rate of absorption of loop diuretics. Hence, peak response arises 4 hours or more after the dose has been administered [23]. Furosemide has a variable oral absorption from 10% to 100% while bumetanide and torsemide have closer to 100% absorptive capacity [12, 22]. Studies show that patients suffering from heart failure (New York Heart Association (NYHA) class II or III) have 1/3rd to 1/4th the natriuretic response to maximally effective dose of loop diuretics. Administering moderate doses at decreased intervals can elevate the response. However giving large doses causes no change in response [12].

Loop diuretics are administered by a threshold type dose-response curve. Furosemide is started with 20 mg and can be incremented up to 40 mg according to the diuretic response. Maximum single oral doses of furosemide for patients with normal glomerular filtration range from 40 to 80 mg and the maximum daily dose is 600 mg. If maximum dose has already been given, it is recommended to increase the frequency of the dose to 2 or 3 times a day. Bumetanide is given at a dose ranging from 2 to 3 mg per day (initial oral dose: 0.5 to 1.0 mg, maximum dose: 10 mg per day) while torsemide is given at 20 to 50 mg per day (initial oral dose: 5 to 10 mg, maximum: 200 mg per day) [24, 25].

Several studies [26–29] have provided evidence that torsemide and bumetanide are more effective than furosemide in the treatment of heart failure. These agents showed superiority in reducing symptoms such as dyspnea and fatigue and resulted in an increased weight loss. Significant decrease in the rates of hospital readmissions and all-cause mortality was also seen [26–29]. These results can be attributed to the higher bioavailability of torsemide and bumetanide over furosemide as described above. Torsemide, in addition, has a longer half-life than both furosemide and bumetanide [26].

Intravenous diuretics are considered to be more potent than oral doses and are used in advanced heart failure. Furosemide is initially administered at a dose ranging from 20 to 40 mg or up to 2.5 times the previously unsuccessful oral dose. In case of a lack of response, the dose can be doubled and repeated at 2-hour intervals till maximum allowed dose levels are reached. The maximum intravenous doses in patients with normal glomerular filtration are 160 to 200 mg of furosemide, 20 to 40 mg of torsemide, or 1 to 2 mg of bumetanide. If a patient has renal dysfunction, higher maximum bolus doses are recommended: 160 to 200 mg of furosemide, 100 to 200 mg of torsemide, or 4 to 8 mg of bumetanide [25].

A Cochrane meta-analysis of 8 trials (254 patients) demonstrated poor evidence to confer supremacy of continuous infusion of loop diuretics over bolus injection in congestive heart failure patients. The results showed an insignificant increase in diuretic effect and better safety profile of the continuous infusion form [39]. A recent single-center, pilot RCT showed that continuous furosemide infusion could lead to better diuresis and greater reduction in b-type natriuretic peptide (BNP) levels for inpatients as compared with bolus injections of furosemide. Nonetheless, the continuous infusion was associated with worsening renal function, longer hospitalizations, and higher rates of adverse episodes during follow-up [40]. A recent large review and meta-analysis (10 trials, 518 patients) showed meaningful differences in neither the efficacy nor the safety of continuous infusion of loop diuretic compared with bolus injections in patients with acute decompensated heart failure [41]. Another meta-analysis to resolve the disparity in previous studies was done, which included 18 RCTs (936 patients). Results failed to exhibit a significant increase in diuresis with the continuous infusion form. However, this review described that, by administering a loading dose and following it up with continuous loop diuretic infusion, a substantial diuresis is achieved in hospitalized patients [42]. All trials and reviews agreed that further, larger studies are warranted to examine if the explored benefits can convert into improved clinical outcomes [39–42].

Combination diuretic therapy (CDT), comprising of loop plus a thiazide diuretic, is recommended for overcoming diuretic resistance in patients with severe volume overload, refractory to adequate dosage (IV furosemide, 160 to 320 mg per day) of intravenous loop diuretic [30]. This approach produces diuretic synergy via “sequential nephron blockade.” Thiazide diuretics block distal tubule sodium reabsorption and can thereby antagonize the renal adaptation to chronic loop diuretic therapy. This improves diuretic resistance secondary to rebound sodium retention. The use of CDT has been shown to result in weight loss, symptomatic improvement, decrease in systemic congestion, hospital discharge, and prevention of readmission. However, careful inspection and frequent monitoring of electrolytes and renal function tests is essential with initiation of CDT as this therapy can lead to severe hypokalemia. Metolazone at a starting dose of 2.5 mg daily is advised for 2 to 3 times weekly dosing in outpatient setting. A 10 mg initial daily dose of metolazone is suggested for inpatients with a 3-day limit to the drug course [30].

Clinical trials such as the Randomized Aldactone Evaluation Study (RALES trial) and the Eplerenone Post-Acute Myocardial Infarction Heart Failure Efficacy and Survival Study (EPHESUS trial) have demonstrated benefits of using aldosterone antagonists (spironolactone or eplerenone) in addition to loop diuretics [43, 44]. The RALES trial demonstrated a 30% reduction in all-cause mortality, with a mean spironolactone dose of 26 mg per day and a 35% reduction in hospitalization for heart failure [43, 45]. In a patient suffering from decompensated heart failure with fluid overload who shows resistance to loop diuretics, natriuretic doses of aldosterone antagonists (spironolactone 50 to 100 mg per day) can be considered as an option [46]. The EPHESUS trial also showed the benefit of eplerenone in decreasing morbidity in dose ranging from 25 to 50 mg per day in patients with heart failure after an acute myocardial infarction and left ventricular systolic dysfunction [44]. One meta-analysis (8 trials, 3929 patients) ascertained that the additional use of an aldosterone antagonist (spironolactone, eplerenone, or canrenone) in treating chronic heart failure patients (NYHA class I to II) reduces mortality and rehospitalization rates and improves heart function with the reversal of left ventricle remodeling [47].

Another positive approach towards diuretic resistant heart failure is the combination of intravenous high-dose loop diuretics with hypertonic saline solutions. Studies have shown this treatment to be efficacious as well as well-tolerated (serum creatinine <2.5 mg/dL) providing symptomatic relief as well as decreasing rehospitalization and long-term mortality [31, 48, 49]. One RCT enrolled 170 patients with refractory congestive heart failure (NYHA class IV) who were unresponsive to high-dose oral furosemide. Treating these patients with intravenous infusion of furosemide (500 to 1000 mg) plus hypertonic saline solution (150 mL of 1.4%–4.6% NaCl) twice a day in 30 minutes showed better daily diuresis and natriuresis in addition to improvement in the quality of life through the relief of signs and symptoms of congestion. Long-term benefit in reduction of mortality rate was also observed when compared with the group receiving intravenous bolus of furosemide (500 to 1000 mg) twice a day, without hypertonic saline solution (55% v 13% survival rate) [31].

Effectiveness of V2 receptor antagonists to treat water retention and hyponatremia in severe heart failure is encouraging. Conivaptan can be used parenterally for inpatients for 4 days while tolvaptan is administered orally for the first day to treat hyponatremia and serum sodium levels are monitored every 6 to 8 hours [1]. The use of tolvaptan may be an effective alternative in the short-term but its use may be limited by its price [48]. Evidence also suggests that tolvaptan can effectively correct chronic hyponatremia for as long as 2 years with minimal side effects (increased urination, thirst) [1, 50].

## 3. Use of Diuretics in Cirrhosis with Ascites

The usually advised first-line therapy includes sodium restriction to 88 mmol/d (2000 mg sodium per day) [51]. Oral diuretics and total abstinence from alcohol are both considered the second line of treatment [11]. Spironolactone is the first-line diuretic recommended for a patient with cirrhosis and edema, initiating with a dose of 50 mg. With its long half-life, doses are altered after 3 to 4 days. Maximum titration sometimes requires higher doses, up to 400 mg per day. However, this may cause gynecomastia [11, 12]. Spironolactone when used alone was as effective as its combined therapy with furosemide [32]. Amiloride can be used as an alternative, initiating with 5 mg per day and titrating up to 20 mg per day. However it is not as effective as spironolactone [51].

In case of an inadequate response to spironolactone, thiazide diuretics are added to the regimen. Depending upon the patient's renal status, doses of 40 mg per day to a maximum of 160 mg per day can be used. Thiazides are terminated if the patient does not respond after 3 days and replaced with a loop diuretic. In patients with renal impairment, frequent doses of moderate amounts are preferred instead of a single large dose. If results are not desirable, spironolactone and thiazides may be added to the regimen. Dietary salt restriction should be ordered in all patients [11, 12].

In comparing the efficacy of furosemide and spironolactone in a randomized comparative study, the activity of the renin angiotensin system proved to alter the action of these diuretics. Patients with high renin and aldosterone levels failed to respond to furosemide but were successfully treated with 300 mg per day of spironolactone [52]. When compared to furosemide, the long acting torsemide produced greater urinary output [53]. Similar results were obtained in an RCT conducted over 70 days with torsemide when compared to furosemide [54]. Administration of octreotide in combination with diuretics not only suppressed both plasma glucagon levels and renin angiotensin system, thereby improving portal and systemic hemodynamics [55].

In a prospective cohort study, human serum albumin was administered to patients who had serum albumin concentration less than 3.5 g/dL and were being treated with furosemide and spironolactone. The body weight loss recorded was dependent upon the amount of human serum albumin administered instead of the dose of diuretics [56]. A reduction in plasma renin concentration was observed in patients treated with human serum albumin combined with diuretic therapy [57].

Combined therapy of 200 mg per day of potassium canrenoate and 50 mg per day of furosemide was more effective when compared with sequential therapy in patients with moderate ascites. Complications such as hyperkalemia were more profound in patients being treated with sequential therapy [58]. Potassium canrenoate and spironolactone are both in the aldosterone antagonist family, having a common metabolite called canrenone spironolactone is more potent and has in addition sulfur-containing metabolites, which have a high renal clearance, thereby allowing access to their site of activity via the renal tubular fluid [59]. For treatment of tense ascites in hospitalized patients, therapeutic paracentesis along with plasma expanders has replaced diuretic therapy and results in fewer complications. However, maintenance diuretics must be given afterwards to prevent recurrence [60].

## 4. Use of Diuretics in Nephrotic Syndrome

Nephrotic syndrome is defined by the presence of proteinuria, edema, hyperlipidemia, and hypoalbuminemia. The incidence of nephrotic syndrome is about 3 new cases per 100,000 each year in adults [33]. Besides the management of underlying disease, treatment of nephrotic syndrome includes limiting proteinuria and inducing diuresis to reduce fluid overload. The key to effective treatment is to create a negative sodium balance. Patients are asked to restrict their dietary sodium intake (<100 mmol per day; 3 g per day), restrict their fluid intake (1.5 liters per day), and take diuretics. Edema should be toned down gradually by avoiding vigorous diuresis that may lead to electrolyte disturbances, acute renal injury, and thromboembolism secondary to hemoconcentration [33].

Due to low serum albumin levels, the diffusion of diuretics in the extracellular compartment is increased. Therefore, a combination of albumin and diuretic may be needed to achieve adequate levels of loop diuretic at the active site. An infusion of 30 mg of furosemide with 25 g of albumin may improve the diuresis. The tubular secretion of furosemide is not affected by this combined therapy. However, this may not apply to patients with serum albumin concentrations of less than 2 g/dL. Therefore, in such patients combined therapies may be theoretically beneficial [61–63]. Moreover, with lesser creatinine clearance, larger doses of diuretic are required to achieve adequate free, unbound drug at the site of action [11, 12].

Coadministration of furosemide with albumin was approved in an RCT with results showing greater urine output and sodium excretion [64]. A meta-analysis revealed that the combination of furosemide and albumin in hypoalbuminic patients demonstrated significant results only within the first 8 hours with respect to greater urine volume and sodium excretion. Results in the next 24 hours were not significant [65]. Another study had conflicting results and concluded that furosemide and albumin combinations should be reserved for diuretic resistant patient with severe hypoalbuminemia [66].

The challenge is to administer the right amount of dose that will reach the active site. The largest dose, also known as the ceiling dose, is an IV bolus of furosemide, 160 to 200 mg or the equivalent of bumetanide and torsemide. Administering such doses yields maximum results however it must be noted that maximum effect is only 20% of filtered sodium [11].

There is evidence indicating that the addition of thiazides with loop diuretics increases overall effectiveness [11]. Metolazone-furosemide combination of diuretics was compared with the thiazide-furosemide combination and it was concluded that similar results occurred with both combinations [67]. Choice of combination diuretics depends highly upon the pharmacokinetics of the drug. Metolazone's action does not differ much from thiazides except for the fact that its elimination half-life is much longer at up to 2 days [11]. In a study assessing the long-term effect of metolazone in patients with nephrotic syndrome, loss of edema and improved control of blood pressure was observed. Moreover, addition of furosemide enhanced diuresis [68].

In a prospective study assessing treatment of children with severe edema and nephrotic syndrome, diuretics were used alone in patients with volume expansion contrary to the regimen of diuretics with albumin in patients with volume contraction. The rationale was the fluid overload associated with albumin administration. Patients with volume expansion were given IV furosemide at 1 mg/kg per dose up to 40 mg twice daily and oral spironolactone at 2.5 mg/kg per dose divided twice daily up to 100 mg. The study concluded that treatment with diuretics alone in pediatric age group was safe and effective [69]. Three children were given a combination of mannitol and furosemide, which led to promising results of 10–30% weight reduction and edema in a week [70].

## 5. Adverse Effects of Diuretics

Thiazide diuretics are known to cause hypokalemia that may result in arrhythmias [34, 35]. The hypokalemic state causes increased blood glucose levels. Correction of potassium levels resolves this glucose intolerance. Thiazides compete with uric acid in renal tubular secretion, which ultimately precipitates hyperuricemia. This state can be managed by taking uric acid lowering drugs such as allopurinol along with thiazides [36]. Loop diuretics are known to cause interstitial nephritis and skin reactions. Loops have to be carefully monitored, especially in high doses as they can precipitate transient ototoxicity. Administering loop diuretics is also associated with hypokalemia, which could cause cardiac arrhythmias and lead to mortality [37]. Loop and thiazide diuretics deplete the body of not only potassium but also magnesium. Their synergistic use results in even further losses of these cations. Oral supplementation and/or potassium-sparing diuretics are used to recover from these losses [11, 12]. The mineralocorticoid receptor antagonist spironolactone, but not eplerenone, can result in gynecomastia [11, 12]. Increased incidence of hyperkalemia was observed with spironolactone. Gastrointestinal side effects and gynecomastia were more pronounced when a combination of spironolactone and furosemide was used compared to a combination of amiloride and furosemide [38].

**Figure 2:**
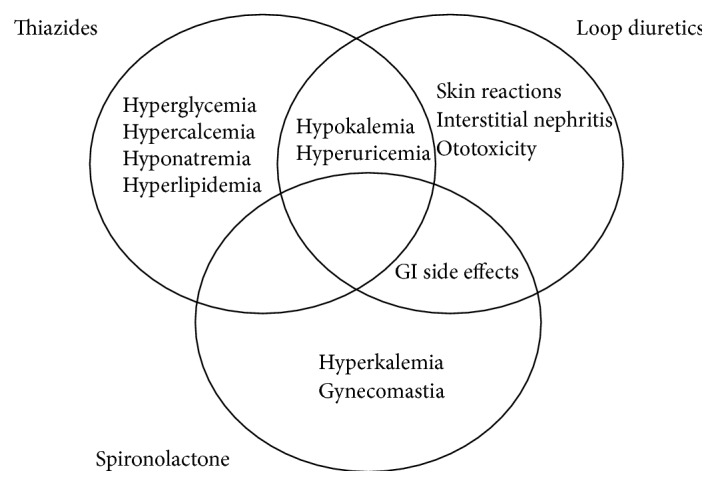
Adverse effects of major diuretics [11, 12, 34–38].

Figure 2 illustrates the common side effects of the main diuretics.

## 6. Conclusions

Effective and adequate diuresis can be achieved in patients with cardiac failure, cirrhosis, and nephrotic syndrome with ideal therapeutic approach of diuretics therapy. Therapy should be directed first to the primary disease mechanism and later to the patient [11, 12]. Each underlying disorder influences the action of the diuretic being administered; therefore, correct choice of drug is essential for successful management [12].
